# Zearalenone and Reproductive Function in Farm Animals

**DOI:** 10.3390/ijms9122570

**Published:** 2008-12-11

**Authors:** Fiorenza Minervini, Maria Elena Dell’Aquila

**Affiliations:** 1 Institute of Sciences of Food Production (ISPA), National Research Council (CNR)/ Via G. Amendola 122/O, 70125 Bari, Italy; 2 Department of Animal Production, University of Bari / Strada Provinciale Casamassima Km3, 70100 Valenzano, Italy. E-Mails: e.dellaquila@veterinaria.uniba.it

**Keywords:** Zearalenone and its derivatives, farm animals, *in vivo*, *in vitro*

## Abstract

Farm animals are exposed to zearalenone through the feed because of the widespread occurrence of this mycotoxin in cereals and clinical reproductive disorders due to mycotoxin effects are often reported in farm animal species. This review describes the metabolism, the mechanistic aspects, the clinical reproductive symptoms and the *in vitro* effects on functional parameters of oocytes and sperm cells induced by zearalenone and its derivatives in farm animals. The studies on *in vitro* effects allow to understand the action mechanisms of mycotoxins and, sometime, to explain the *in vivo* symptoms. The impairment of semen quality and female reproductive function induced by zearalenone could be a factor responsible for the reproductive failure in farm animals.

## 1. Introduction

**Figure 1.**
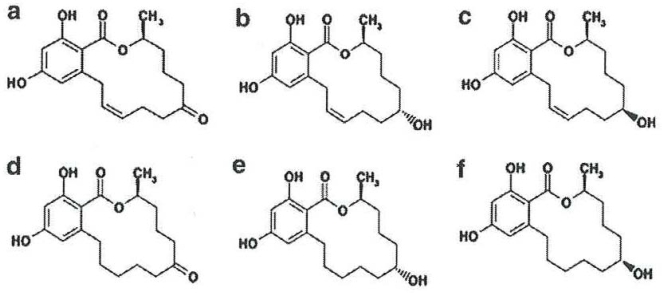
Chemical structures of zearalenone and its derivatives: a) zearalenone (ZEA), b) α-zearalenol (α-ZOL), c) β-zearalenol (β-ZOL), d) zearalanone (ZAN), e) α-zearalanol (α-ZAL), f) β-zearalanol (β-ZAL) [1].

Zearalenone (ZEA) is a mycotoxin produced by *Fusarium graminearum*, *F. culmorum*, *F. crookwellense*, *F. equiseti* and *F. semitectum*, frequent contaminants of maize, wheat, oats and barley [1]. This mycotoxin may co-exist with deoxynivalenol (DON), as the same fungi, *F. graminearum* or *F. culmorum*, may produce both compounds [1]. In mammals, the keto group at C-8 is reduced to two stereoisomeric metabolites of ZEA (α- and β-isomers) (Figure 1). The ZEA metabolites, such as α-zearalenol (α-ZOL), β-zearalenol (β-ZOL), α-zearalanol (α-ZAL), β-zearalanol (β-ZAL) and zearalanone (ZAN), are also produced by the fungi, as reported by Bottalico *et al.* [2] in corn stems infected with *Fusarium*. Variations in the incidence of ZEA occur with different crop years, cereal crop and geographical areas. The worldwide contamination of grain and feed with ZEA ranges from 0.004 to 8 mg/Kg, with the highest levels found in wheat (from Germany) and corn samples (from Argentina) [3]. Considering the mean levels of ZEA in the principal foods and their consumption, the average human daily intakes of ZEA ranges among adults from 0.8 to 29 ng/Kg bw., while small children have the highest average daily intakes ranging from 6 to 55 ng/Kg/b.w./day [4]. The great variability in diet composition for the major farm animal species precludes the calculation of actual exposure levels based on the occurrence of ZEA in individual feed materials, considering also the ZEA occurrence in straw, hay, pastures or grass-clover less documented [5].

## 2. Metabolism of zearalenone in farm animals

Zearalenone is rapidly absorbed after oral administration. Its uptake is estimated to be 80-85% and the mycotoxin and its derivatives are detected in blood about 30 min after oral administration [6] bound to human globulins, as reproductive hormones [7]. Studies of the metabolic route and pattern after ZEA ingestion explain the cause of differences between farm animals and its action mechanism [6]. According to Olsen [6], two major biotrasformation pathways for ZEA in animals have been suggested:

1. Hydroxylation resulting in the formation of α- and β-ZOL, catalyzed by 3α- and 3β- hydroxysteroid dehydrogenase (HSDs);
2. Conjugation of ZEA and its reduced metabolites with glucuronic acid, catalyzed by uridine diphosphate glucuronyl transferase (UDPGT).

The liver is the main organ responsible for metabolism of steroids, but a variety of other tissues, such as kidneys, testis, prostate, hypothalamus, ovary, intestine, contain 3α(β)-HSD activity. The adverse effects of ZEA are partly determined by the processes of elimination, because the biliary excretion and entero-hepatic cycling are important processes affecting the fate of ZEA [8] and explaining a different sensitivity between animals.

### 2.1. Pig

Malekinejad *et al.* [9] demonstrated that in a hepatic biotrasformation pigs convert ZEA predominantly into α-ZOL. An extrahepatic biotransformation of ZEA into α-ZOL was reported in porcine granulosa cells by means of 3 α-HSD [10]. In the sow, it has been reported that the intestinal mucosa has a very active glucuronide conjugation (about 30 times) of ZEA compared with the levels of reduction. It seems likely that ZEA is conjugated before it is reduced to zearalenol [6]. The glucuronide of ZEA is excreted in bile to be re-absorbed and metabolized further by intestinal mucosal cells (manly in α-ZOL), ultimately entering the liver and the systemic circulation via the portal blood supply [8]. The entero-hepatic cycle has the effect of prolonging the retention of ZEA and its derivatives in the circulatory system, retarding elimination and enhancing the duration of adverse effects [8]. After a low-dosage feeding (192 μg/Kg b.w./day for 4 day) of prepubertal gilt, α-ZOL was detected in plasma one hour later at concentration twice (2 ng/mL) as high as ZEA and rose to 3-4 times (8 ng/mL) during the day of ZEA feeding [11]. The maximum blood levels of both mycotoxins (33,000 pg/L) are 500 times higher than the concentration of 17β-oestradiol (17βE_2_) at oestrous but they are bound to glucuronic acid, which inactivates the compound and facilitates the excretion [11]. The urinary concentrations of ZEA and α-ZOL reach maximum level (158.9 ng/mL and 170.8 ng/mL, respectively) in the end of ZEA feeding and both compounds could be traced 4 days after [11]. Higher blood levels in gilt and sows (from 3 to 100 ng/mL), in wild boars (from 2.2 to 43 ng/mL) and foxes (from 5 to 145 ng/mL) were found in Poland by Gajecki [7] respect to concentrations (up to 0.96 ng/mL) in Romanian slaughtered pigs [12].

### 2.2. Ovine

The ovine metabolism of ZEA includes the synthesis of five metabolites, such as ZAN, α- and β-ZOL, α- and β-ZAL and high levels of some of these forms may be excreted in the urine as glucuronides by grazing sheep [8]. As reported by Malekinejad *et al.* [9], the sheep liver post-mitochondrial fraction converts ZEA mainly into α-ZOL. The concentration of α- + β-ZOL, found by [13] in urine collected from pasture-fed animals, resulted higher (up to 86 ng/mL) than that of α- + β-ZAL (up to 2.1 ng/mL).

### 2.3. Bovine

It has been reported that bovine hepatocytes and granulosa cells (GCs) produce predominantly β-ZOL [6, 9, 10] that was found as the predominant metabolite in urine and faeces from a cow fed with zearalenone. As reported by Erasmuson *et al.* [13], high amounts of α- + β-ZAL (up to 12.3 ng/mL) and α- + β-ZOL (up to 163 ng/mL) were found in urine samples (68%) of pasture-fed cattle.

### 2.4. Horse

The main excretion of ZEA in the horse come through faeces, followed by urine [14]. The main metabolites detected in the equine faeces are ZEA (73 ng/mL), α-ZOL (50 ng/mL) and β-ZOL (45 ng/mL). α-ZAL (27 ng/mL), β-ZAL (41 ng/mL) and ZAN (29 ng/mL) were found at low levels. Zearalenone urinary levels of 3 ± 2 (mean ± standard deviation) and 43 ± 57 ng/mL were found in Italian and Romanian horses, respectively, as natural exposure to ZEA [15]. Erasmuson *et al.*[13] reported very high levels of α - + β-ZOL (up to 19 ng/mL) and α -+ β-ZAL (2157 ng/mL) in urine samples probably related to the grazing practices for those species.

## 3. Action mechanisms of zearalenone and its derivatives

### 3.1. ER-related effects

Zearalenone and its derivatives compete effectively with 17 β-E_2_ for the specific binding sites of the oestrogen receptors (ERs) occurring in different organs. Two subtypes of ER exist, ER- alpha and ER-beta that are differently distributed in the body. Several investigations have demonstrated that binding of ZEA and its derivatives initiate a sequence of events known to follow estrogen stimulation of target organs [16]. In the uterus, the cytoplasmic receptor complex is translocated to the nucleus (with a time retention longer that E_2_) and the following events, typifying early estrogen response, occur: an increase in uterine RNA synthesis as well as an increase in RNA polymerase activity, synthesis of uterine estrogen-induced protein [16]. The binding of ZEA to ER in target tissues is < 1–10% than that of E_2_, whereas α-ZOL shows somewhat stronger binding and β-ZOL much less binding [17]. The relative binding affinities to the rat uterine cytoplasmic receptor for ZEA and derivatives are α-ZAL>α-ZOL> β-ZAL>ZEA>β-ZOL [17]. The competition of binding to ERs is demonstrated with *in vitro* systems. The proliferation of ER-positive (MCF-7) and ER-negative (MDA-MB-231) human breast cancer cells lines, which respond to physiological concentrations of E_2_ (1nM) was used to characterize the oestrogenic activity of ZEA and derivatives through oestrogenic parameters such as proliferative effect (PE), relative proliferative effect (RPE) and relative proliferative potency (RPP) [4]. On MCF-7, α-ZOL induced a higher PE and RPP, probably ER α–mediated, (because of partial antagonism of mycotoxin-related proliferation by tamoxifen). The potency of ZEA and derivatives were lower (from 10^−2^ to 10^−3^) than E_2_ [4]. β-ZOL showed higher EC_50_ value (5.2 × 10^−3^ μM) than those of other ZEA-related compounds [4]. ZEA and its derivatives rapidly enhance phasic spontaneous smooth contraction in myometrial strips of prepubertal lamb at nanomolar concentration like E_2_. Both intracellular ER and plasma membrane receptors could be involved in this effect [18]. In fact, referring to myometrium, the existence of novel E_2_ membrane binding proteins, structurally related to the intracellular ER, has been previously described in rabbit [19]. The involvement of non-genomic actions is observed especially for ZEA, which increases the uterine activity similarly to E_2_. An antiestrogenic effect is found for α-ZOL, which can be based on the fact that α-ZOL blocks ER on cell membrane following no biochemical response [18].

### 3.2. Hormonal-related effects

**Figure 2.**
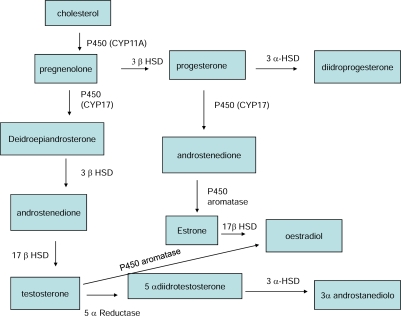
Biochemical pathway from cholesterol to steroid hormones.

The structure of the ZEA resembles many characteristics of steroids and binds to ER as an agonist. The ZEA conversion to α-ZOL and β-ZOL shows similarities to processes in steroid metabolism where HSDs play a pivotal role in synthesis of various steroid hormones including E_2_ and testosterone. Consequently, ZEA is also a substrate for 3α-HSD and 3β-HSD present in many steroidogenic tissues, such as liver, kidney, testis, prostate, hypothalamus, pituitary, ovary, intestine [6]. As observed in Figure 2, the enzymes involved in the biosynthesis of the gonadal steroid hormones (such as progesterone, E_2_ and testosterone) fall into two major classes of proteins: the cytochrome p450 heme-contaning proteins and the hydroxysteroid dehydrogenases (HSD) [20,21,22]. The first and rate limiting step in the synthesis of steroid hormones is the conversion of cholesterol to pregnenolone, catalysed by the enzyme CYP11A (cytochrome p450_scc_) found in the ovary (theca interna and GCs) and testis (Leydig cells). The CYP17 is expressed exclusively in the Leydig and in thecal cells where there is the site of androgen production. Cytochrome p450 aromatase catalyses the conversion of androgen to estrogens in two different major pathways: first, the aromatase pathway (transformation of testosterone into E_2_ and of androstenedione into estrone) and secondly, the 5α-reductase pathway (transformation of testosterone into dihydrotestosterone). The major sites in human and rats are in the preovulatory follicle, the corpus luteum, placenta and Leydig cells. 3α-HSD shows high affinity for 5α-dihydrotestosterone. The seven human isoenzymes of 17β-HSD play an important role in the biosynthesis of mainly androgens and oestrogens, testosterone and estradiol and their weaker precursor androstenedione and estrone which are expressed in GCs of developing follicles and in testes (Leydig cells). The 3 β-HSD catalyses the conversion of pregnenolone into progesterone. The isoforms of 3 β-HSD are expressed in a cell- and tissue-specific manner, particularly in ovary (thecal and GCs of growing and preovulatory follicles) and testis (Leydig cells).

The conversions of testosterone and progesterone by these tissues are important components of the mechanisms by which these hormones exert their effect on both gonadotropin regulation and sexual behavior. By competitively inhibiting the 3α-reduction of these hormones, ZEA could cause an accumulation of the active components, because 3α-HSD is an important factor in ovarian follicular development [6]. Zearalenone interference with HSDs may affect the reproduction, in addition to the effect via ER.

*In vitro* studies on mouse Leydig cell function demonstrated that ZEA and α-ZOL exposure (from 10^−8^ to 10^−4^ M) can interfere with the process of spermatogenesis reducing the hCG-stimulated testosterone synthesis owing to the down-regulation of P450scc, 3β-HSD-1 and steroidogenic acute regulatory protein (StAR) transcription [23].

## 4 *In vivo* exposure of zearalenone and its derivatives

The effect of ZEA and its metabolites depends upon the reproductive status (prepubertal, cycling or pregnant) of the affect animals.

### 4.1. Pig

The specific manifestations are dependent upon the relative dose of ZEA and the stage of estrous cycle or pregnancy during which ZEA was consumed. The oestrogenic syndrome in swine affects primarily the reproductive tract and mammary gland, and in more sensitive young gilts 1–5 ppm of ZEA induce clinical signs such as hyperemia, edematous swelling of the vulva, sometime vaginal prolapse and even rectal prolapse [24]. This hyperoestrogenic mycotoxicosis primarily affects weaned and prepubertal gilts because the lack of full development of the endogenous endocrine system in the young [24]. In immature gilts, dose of 200 μg/Kg b.w. leads to the development and maturation of the largest follicles through the activation of an apoptotic-like process in the granulosa layer of single mature follicle and ovarian follicle atresia [25, 26]. After recovery of oocytes during ovario-hysterectomy of gilts exposed to ZEA and DON by feeding (from 0.2 and 0.004 to 9.6 and 0.36 mg/Kg feed, respectively), oocytes with compact cumuli showed a significantly reduced proportion with immature chromatin in groups exposed to high mycotoxin levels [27]. The proportion of oocytes having degenerated meiotic chromatin was significantly higher in these groups compared with groups exposed to lower concentrations. The oocyte quality was significantly reduced and was directly related to the reduced proportion of normal germinal vesicle-stage oocytes [27]. The feeding of wheat contaminated naturally with DON and ZEA to gilts did not influence the activity of enzymes (P450scc and 3β-HSD) involved in progesterone synthesis, as observed *in vivo*-derived porcine granulosa cell cultures [27]. Oocyte degeneration and reduced meiotic competence of compact cumulus oocyte complexes were reported after *in vitro* maturation [27].

In cyclic animals, nymphomania, pseudopregnancy, ovarian atrophy and changes in the endometrium are reported [24]. Zearalenone causes sterility in sows by inciting a malfunction of the ovary [28]. The oocyte dies in the Graafian follicles and despite signs of oestrus, there is no ovulation. In gilts, after 100 ppm in feedstuff for one week of treatment, ZEA appears to cause maturation by stimulating primary and secondary follicles in the ovary and proliferation of uterine glands [28]. Zearalenone acts similarly to E_2_ in inhibiting the release and secretion of follicle stimulating hormone (FSH), thus depressing the maturation of ovarian follicles during the preovulatory stage. The changes induced by ZEA depend on time of administration in relation to oestrous cycle as well as on the dose administered [24]. This indicates that ZEA may exhibit a luteotrophic property which prolongs the life span of the corpus luteum equal to or longer than the normal gestation period [24].

During pregnancy, the timing of the exposure is critical, because at threshold level of ZEA (1 mg/Kg b.w.), administered on days 7 to 10 after mating, does not disrupt pregnancy, when administered on days 2 to lead to degenerative changes in embryos that begin to be well advanced by day 13 after mating [29]. These effects are similar to those of other exogenous estrogens on pregnancy, such as E_2_. During pregnancy, ZEA reduces embryonic survival, when administered a threshold level (200 μg/Kg b.w.), and sometimes decreases fetal weight [8]. Placental transfer of ZEA results in teratogenic effects in piglets characterized by various abnormalities of genitalia [8].

The effect of *Fusarium* cultures *per os* on male swine showed a 30% of weight reduction of testes, decrease of fertility related to reduction of sperm quality and viability [28]. In boar, ZEA depressed serum testosterone, inducing feminization and suppressing libido [8]. No adverse effect on the reproductive potential of mature boars was described at levels normally found in contaminated feed, but in female swine several severe reproductive problems can occur after feeding with ≤200 ZEA mg/Kg [30]. The α-ZOL may reduce aggressive behavior, testis growth and sexual activity in farmed fallow bucks by ear implants at a dose of 36 mg at 90 day intervals [31].

### 4.2. Bovine

In cow, infertility, reduced milk production and hyperoestrogenism have been associated with ZEA [8]. Heifers were fed with ZEA containing diet over three estrous cycles, conception rates decline from 87 to 62% [8].

#### 4.2.1 Ovine

High dietary levels (12 mg/Kg of diet) of ZEA feeding for extended periods of time (10 days) may affect reproductive performance of sheep negatively by reducing fertility and ovulation rates [32]. A daily intake of 12 mg ZEA for eight weeks during the breeding season did not affect the weekly sperm production, spermatozoal mass motility of spermatozoal morphology of adult ram [33].

### 4.3. Horses

A field outbreak of ZEA mycotoxicosis in horses was associated with corn screenings containing approximately 2.6 mg/Kg of ZEA [34]. The concentration of the toxin recorded in naturally contaminated oats (ZEA and DON at 1 and 12 ppm levels, respectively) does not have a significant effect on the release of reproductive hormones, cycle length or uterine histology in mares [35]. Local ovarian effects of both mycotoxins on follicular growth cannot be excluded because the number of growing follicles during the second half of the cycle were increased [35]. Zearalenone, administered at low dose (7 mg) starting 10 days after ovulation, did not influence the interovulatory interval, the length of luteal and follicular phase, plasma progesterone concentration, the number of large follicles (>2cm) [36]. Outbreak of early fetal losses, abortions, stillbirths and neonatal foal deaths (collectively referred to as the Mare Reproductive Loss Syndrome MRLS) were reported in North-Central Kentucky during the spring of 2001. Several feed-related causes, such as infestation with caterpillar larvae and/or natural toxic compounds (cyanogens, ergot alkaloids and mycotoxins) were hypothesized, but MRLS was not linked to mycotoxins [37].

## 5. *In vitro* exposure of zearalenone and its derivatives

As reported in Table 1, most of the alterations in the reproductive tract caused by ZEA exposure have been reported as a result of *in vivo* investigations which provide information about net effects in whole animals, where pollutant may act at multiple sites and organs but these studies reveal little about the involved underlying mechanisms [38]. Therefore, cell and tissue cultures are of increasing importance for the evaluation of the risks due to these toxic compounds [39]. In fact, the knowledge of the structure-function relationships in cell culture could be utilized to predict the mycotoxin toxicity in *in vivo* systems, however these systems not always provide an accurate prediction of toxicity in whole animals [38].

### 5.1. Follicle development, oocyte maturation and embryo development

#### 5.1.1. Sow

*In vitro* maturation (to telophase I and metaphase II; TI and MII) of porcine oocytes is negatively affected by α- and β-ZOL in a dose-dependent manner [38]. Culture of oocytes in the presence of α-ZOL for 48 h up to the concentration of 7.5 μM resulted in a significant decrease in the maturation rate, whereas β-ZOL showed a significant effect only at 30 μM [38]. The development of *in vivo*-produced porcine zygotes to blastocysts was influenced by α-ZOL. At concentration of 7.5 μM, the percentage of zygotes developing to blastocysts tended to be reduced. Increasing concentrations of mycotoxins in the culture medium increased the degeneration of embryos [38]. Similarly to E_2_, ZEA and its derivatives (α- and β-ZOL) at levels ranging from 0.3 to 31.2 μM, reduced the percentage of oocytes that reached the MII stage, induced nuclear malformation in a concentration-dependent manner, with ZEA and α-ZOL being the most effective at lower concentrations. All mycotoxins reduced the fertility by altering spindle formation during meiosis and leading to less fertile oocytes and mixoploid embryos [40]. Alpha and β-zearalenol, at levels of 15 and 30 μM, reduced the FSH or foskolin enhanced basal progesterone synthesis in primary cultures of porcine GCs by reducing the expression of cytochrome P450scc and 3β-HSD transcripts [41]. On porcine GCs α-ZOL had a biphasic effects on IGF-I-induced estradiol production, increasing E_2_ production at lower levels (from 0.094 to 0,936μM) and inhibiting at larger doses (9.36 μM), as reported by Ranzenigo *et al.* [42]. At highest concentration α-ZOL increased IGF-induced progesterone production and at lower levels (from 0.094 to 0,936μM) increased FSH-induced progesterone production [42].

#### 5.1.2. Cattle

Zearalenone and its derivatives (α-ZOL and ZAN) inhibit *in vitro* maturation of oocytes to the M II stage and increase the rates of oocytes showing chromatin abnormalities at levels of 94 μM [43]. This effect is dose-dependent and a trend of increasing maturation rates, inversely related to mycotoxin concentration, is observed. When toxins were added at levels of 0.94 or 9.3 μM, most of the oocytes, which did not complete maturation, were found as blocked at stages comprised between M I and T I [43]. After *in vitro* exposure of mural GC cultures with 94 μM of α-ZOL for 24 h, an increase of 17β-E_2_ levels was found in culture medium supernatants, probably related to the inhibitory effects of mycotoxins in the pathway of steroidogenesis [43].

#### 5.1.3. Mare

The *in vitro* exposure of GCs collected from the ovaries of cycling mares with ZEA and its derivatives α- and β-ZOL, at levels of 1 and 0.1 nM, induced a simultaneous increase in cell proliferation and an apoptotic process [44]. The contemporaneous occurrence of both processes could indicate that these mycotoxins could be effective in inducing follicular atresia and these effects may result from both direct interaction with ERs as well as interaction with the enzymes 3- α- (β-)HSD present in the ovary and GCs and involved in the synthesis and metabolism of endogenous steroid hormones [44].

### 5.2. Spermatozoa parameters

#### 5.2.1. Boar

Zearalenone and α-ZOL affect the fertilization ability of boar sperm because of their negative effect on viability, motility and acrosome reaction (evaluated by microscopic observation) in a time and dose-dependent manner after *in vitro* exposure at concentrations ranging from 125 to 250 μM [45]. After 4 h of incubation at lower levels (from 30 to 95 μM) of ZEA and α-ZOL, a boar-dependent chromatin instability is noted by microscopic observations [46]. Mycotoxins induce a mycotoxin-dependent toxic effect on functional sperm parameters after exposure from 2 x 10^−7^ to 20 μM of ZEA and its derivatives (α- and β-ZOL) for 24 and 48 h [47]. Zearalenone is the most cytotoxic at low levels (pM concentrations) followed by α-ZOL (nM levels) and β-ZOL (μM levels) after 24 h of incubation. Sperm motility, evaluated by CASA, is affected by *in vitro* exposure to mycotoxins: in particular β-ZOL significantly increases the curvilinear velocity (VCL parameter) at μM levels, 100-fold lower than those active for α-ZOL, after short (5 h) time incubation and no modifications are observed at longer times [47]. Rajkovic *et al*.[48] report a 10% decrease of percentage progressive motility after 5 minutes exposure to 31.4 mM of ZEA. The DNA integrity and structural stability (evaluated by flow cytometry using acridine orange), strictly related to male fertility and to early embryonic development [49], is modified after α-ZOL exposure at pM levels and β-ZOL at nM levels for 24 h. While longer incubation (48 h) induced a general no-concentration-dependent toxicity for all mycotoxins, observed as a graphical modification to the histogram [47].

#### 5.2.2. Stallion

Zearalenone and its derivatives (α- and β-ZOL; α- and β-ZAL and ZAN), added to Tyrode’s medium at concentrations ranging from 0.025 to 250 nM induce instability of sperm chromatin structure of frozen-thawed spermatozoa at lower levels. β-ZOL, ZEA, α-ZOL and α-ZAL induce chromatin damage at 0.025 nM instead of ZAN and β-ZAL that are toxic at 0.25 nM [50]. The instability of sperm chromatin structure was found after *in vitro* exposure of equine spermatozoa with some urine samples containing low levels (ng/mL) of ZEA and its derivatives measured by ELISA [15]. This effect on chromatin structure, strictly related to subfertility in stallions [51] and early miscarriage/abortion in humans [49], was measured by flow cytometry by using acridine orange.

## 6. Conclusions

The effects of ZEA have been widely investigated *in vivo* rather than *in vitro*, especially in sensitive animal species, such as swine. The *in vivo* investigations provide information on the net effects in whole animals, whereas cell-specific responses emerge from *in vitro* investigation. The summarized results of *in vitro* studies with cell cultures of the reproductive tract indicate that there is only partial agreement with those of *in vivo* studies, because multiple interactions occur in whole organisms during mycotoxin exposure. *In vitro* experiments may contribute to risk assessments and to define the action mechanism induced by mycotoxins on germ cells. *In vitro* effect of ZEA and its derivatives was more investigated in cells of ovaries compared to those of testis. Complications in pharmaco-kinetic distribution and secondary effects attributed to other unidentified factors may make it difficult to ascertain the direct mechanistic toxicities of mycotoxins to the cells To our knowledge, innovative methodologies, such as the Computer Assisted Sperm Analysis (CASA) or flow cytometry, have not been used to assess sperm cell functional parameters during mycotoxicosis outbreaks. Therefore, it is necessary to increase the use of cell models in order to determine the direct biological effects of mycotoxins in order to validate the *in vivo* findings [22].

## Figures and Tables

**Table 1.** Comparative *in vivo* and *in vitro* toxic effects of zearalenone and its derivative in pigs.

*Sow*				
*Target*	Oocyte	Granulosa cells	Hormonal status	Ovary
***In vivo***	Oocytes with degenerated meiotic chromatin [27]		No modification of progesterone synthesis [27] ↓ FSH [24]	Stimulation of primary and secondary follicles [28] Ovarian follicle atresia [25,26] Delay of maturation of ovarian follicles during preovulatory stage [24]
***In vitro***	Alteration of spindle formation during meiosis [39] Delay of TI and MII oocyte stage and nuclear malformation *[8]*	↓ FSH or foskolin- enhanced progesterone [41] Modulation dose- related IGF-induced oestradiol, progesterone and of FSH-induced progesterone [41]		

*Boar*				
Target	Hormonal status	Sperm motility	Sperm viability	Chromatin structure
***In vivo***	↓ testosterone [8]		↓ [28]	
***In vitro***		↓ [44,46]	↓ [44,46]	↓ [44,46]
